# Environmental Risks and Toxicity of Fipronil and Imidacloprid Used in Pets Ectoparasiticides

**DOI:** 10.3390/ani15111533

**Published:** 2025-05-23

**Authors:** Lucia De Marchi, Matteo Oliva, Maria Nobile, Mario Carere, Luca Maria Chiesa, Donatella Degl’Innocenti, Ines Lacchetti, Laura Mancini, Valentina Meucci, Carlo Pretti, Marzia Vasarri, Roberto Edoardo Villa, Luigi Intorre

**Affiliations:** 1 Veterinary Teaching Hospital, Department of Veterinary Sciences, University of Pisa, 56124 Pisa, Italy; valentina.meucci@unipi.it (V.M.); carlo.pretti@unipi.it (C.P.); luigi.intorre@unipi.it (L.I.); 2 Consortium for the Interuniversity Center of Marine Biology and Applied Ecology “G. Bacci”, 57127 Livorno, Italy; oliva@cibm.it; 3 Department of Veterinary Medicine and Animal Science, University of Milan, 26900 Lodi, Italy; luca.chiesa@unimi.it (L.M.C.); roberto.villa@unimi.it (R.E.V.); 4 Department of Environment and Health, Italian National Institute of Health, 00161 Roma, Italy; mario.carere@iss.it (M.C.); ines.lacchetti@iss.it (I.L.); laura.mancini@iss.it (L.M.); 5 Department of Experimental and Clinical Biomedical Sciences, University of Florence, 50134 Florence, Italy; donatella.deglinnocenti@unifi.it (D.D.); marzia.vasarri@unifi.it (M.V.)

**Keywords:** ectoparasiticides, fipronil, imidacloprid, pets, environmental risks, ecotoxicological assays, human health

## Abstract

Fipronil (FIP) and imidacloprid (IMID) are two of the most commonly used ectoparasiticides to control parasites in pets. Compared with those of farm animals, their environmental risks have generally been considered low because of their limited use; however, the growing pet population and evolving treatment practices make this assumption challenging. To assess these risks, water samples were collected at an animal shelter in Italy to monitor the abundance of ectoparasiticides in aquatic environments. Additionally, laboratory-based ecotoxicological assays were carried out on a range of marine non-target species across different trophic levels (algae, copepods, and mussels). In vitro toxicity tests on human epithelial cell cultures were also implemented to examine potential cytotoxic effects at the levels of human exposure detectable in a domestic setting after pet treatment. Wastewater samples from the shelter contained 0.18 µg L^−1^ of IMID, 0.50 µg L^−1^ of FIP, and 0.20 µg L^−1^ of FIP-sulfone, with these concentrations remaining stable for 60 days. Chronic exposure to FIP and IMID at 30.0 µg L^−1^ impaired the mobility of the copepods. The EC_10_ and EC_20_ values were determined to be 1.7 (0.06–6.59) µg L^−1^ and 2.8 (0.436–8.51) µg L^−1^ for FIP and 2.6 (0.80–6.33) µg L^−1^ and 7.6 (3.12–15.8) µg L^−1^ for IMID, respectively. FIP and IMID exposure led to lipid peroxidation in the digestive glands and gills of mussels, whereas only IMID exposure increased acetylcholinesterase activity in the digestive glands at concentrations between 0.5 and 5.0 µg L^−1^. Additionally, both fipronil and imidacloprid triggered the production of reactive oxygen species and lipid peroxidation and decreased the viability of human keratinocyte cells in a concentration-dependent manner. These findings highlight the persistence and potential risks of FIP and IMID, stressing the need for stricter regulations and further research on chronic environmental exposure to safeguard ecosystems and public health.

## Simple Summary

Fipronil (FIP) and imidacloprid (IMID) are common pet treatments for parasites, but their environmental impact is often overlooked. This study monitored their presence in wastewater from an animal shelter in Italy and assessed their effects on marine species and human cells. The findings revealed that FIP and IMID persist in water and can harm aquatic organisms, including copepods and mussels, while also causing toxic effects on human skin cells. These results suggest a need for stricter regulations and further research to understand the long-term environmental and health risks of these chemicals.

## 1. Introduction

Ectoparasiticides are key to protecting the health of companion animals and their owners from ectoparasites and associated diseases [1]. Ectoparasiticides represent the second-largest segment of the global animal health market: parasiticides for companion animals have a market share comparable to that of livestock, and ectoparasiticides account for 49% of the market share [2]. Generally, they offer prolonged efficacy, enabling prophylactic use to prevent reinfestation [3].

Despite the availability of newer compounds such as isoxazolines [4], fipronil (FIP) and imidacloprid (IMID) are still used owing to their efficacy and affordability [5,6]. When topically applied as spot-on formulations, IMID and FIP spread rapidly over the skin via translocation. This process allows the active compounds to cover the animal’s body surface without entering systemic circulation. IMID and FIP become sequestered within hair follicles, sebaceous glands, and skin, where they are gradually released with sebum, thus maintaining prolonged efficacy against parasites.

FIP is a broad-spectrum phenylpyrazole pesticide that controls fleas and ticks in domestic animals through topical formulations, such as sprays and spot-ons [7]. FIP acts on insect gamma-aminobutyric acid (GABA) receptors, where it induces neurotoxicity by blocking GABA-regulated chloride channels, causing hyperexcitation in the insect nervous system. In the environment, FIP degrades through several pathways, such as reduction, oxidation, photolysis, and hydrolysis processes, each resulting in different by-products: FIP-sulfide, FIP-sulfone, FIP-desulfinyl, and FIP-amide, respectively [8,9]. Although FIP is highly selective for insect GABA receptors, its primary metabolite, FIP-sulfone, can still interact with mammalian GABA receptors. Moreover, FIP-sulfone can accumulate in tissues, potentially causing toxic effects in both target and non-target organisms [10].

IMID is a neonicotinoid insecticide that binds to nicotinic acetylcholine receptors (nAChRs). Although neonicotinoids are generally considered safe for mammals because of their greater affinity for insect nAChRs and lower affinity for mammalian nAChRs, IMID degrades into the environment primarily through photolysis, microbial degradation, and hydrolysis into more toxic forms, such as desnitroimidacloprid (DNI) [11]. DNI partially bypasses the insecticide’s intended selectivity, potentially heightening neurotoxic risks even for non-target species [12].

The environmental impact of veterinary medicinal products (VMPs) for companion animals is considered negligible according to regulations given that these animals are not intensively farmed, treatments are administered individually, and overall product use is lower compared to food animals. As a result, VMPs for pets do not require a Phase II Environmental Risk Assessment (ERA), which evaluates the potential long-term environmental impacts and the persistence of the product in various ecosystems under conditions of use [13,14].

However, the rapid growth in companion animal populations, combined with new treatments, has prompted re-evaluation of these assumptions. In 2023, the European Medicines Agency (EMA) released a Concept Paper [1], proposing potential updates to environmental risk assessments (ERAs) for parasiticides used on companion animals, including the IMID and FIP. The paper suggests that both substances may soon be added to a “watch list” owing to their potential toxicity to non-target species. This highlights the inadequacy of current ERA protocols in the changing environmental landscape. Recent findings also indicate that the growing number of treated animals and the increased frequency of product use may have intensified the environmental presence of these parasiticides beyond initial estimates, underscoring the need for a comprehensive reassessment of associated ecological risks [15,16,17].

In a recent paper, Perkins et al. [16] reported that pet ectoparasiticides can reach aquatic environments both via down-the-drain (DTD) pathways, through wastewater, and directly when dogs enter waterways. Studies from the U.S. and the EU have revealed the near-ubiquitous presence of FIP and IMID in wastewater effluents, implicating VMPs as important contributors.

The transfer of FIP to wastewater when bathing spot-on-treated dogs has been demonstrated qualitatively [18,19,20]. Budd et al. [20] provided the first direct evidence that topical parasiticides can be a significant source of wastewater pollution. They reported pesticide concentrations in the outflow from a pet-grooming facility, a laundromat, and a pest control operation in California. Their aim was to assess the relative contributions of these sites to a sewer catchment. Significantly high concentrations of FIP, its metabolites, and IMID were observed at the pet-grooming facility, with relatively minor concentrations at the other sites.

The situation is compounded by the fact that wastewater treatment plants (WWTPs) either do not exist, are outdated or are not designed to remove micropollutants such as VMPs. Conventional WWTPs target organic matter, nutrients, and pathogens but lack ozonation, activated carbon, or advanced oxidation processes needed to remove persistent substances such as FIP and IMID. In rural or economically disadvantaged areas, financial and technical limitations, along with insufficient regulation, often result in untreated or only partially treated wastewater being discharged into the environment [21]. Even advanced plants may not fully eliminate these compounds, thus leading to their widespread occurrence in aquatic ecosystems [22].

Pet ectoparasiticides can also contaminate the home environment, particularly carpets, sofas, and blankets, and residues may persist for days or even weeks, depending on the drug’s chemical composition. Airborne dispersion from sprays and powders can cause the medication to spread and settle on such textile surfaces, and medications absorbed into the pet’s fur can be deposited when the pet rubs itself against furniture or lies down on fabrics. Moreover, after topical medications (e.g., spot-ons, sprays, powders) are administered to pets, active ingredients can be transferred to household surfaces through direct contact.

This indirect exposure poses potential risks to humans, especially young children, individuals with allergies, and those who are immunocompromised [23]. The accumulation of these substances may cause skin irritation or allergic reactions from direct contact with contaminated fabrics. Inhalation of residual particles or accidental ingestion, particularly in young children who play on carpets or put contaminated objects in their mouths, also poses significant risks [17].

Therefore, it is crucial to study the risks of human exposure to ectoparasiticides in the home environment to ensure the safety of pet owners [24,25]. Furthermore, residues that enter domestic and shelter environments can eventually enter aquatic ecosystems, disrupting local biodiversity and harming nontarget species, potentially leading to ecological decline [9,26].

The aim of our study was thus to assess the environmental and ecological impact of FIP and IMID, which are used as ectoparasiticides in companion animals. The concentrations of these chemicals in wastewater from a shelter in Italy were determined following routine animal treatments. Sampling took place at the wastewater collection pit, thus enabling the analysis of water leaving the facility before entering the aquatic environment.

The environmental toxicity of these chemicals was assessed through laboratory-based ecotoxicological assays and biochemical analyses of marine non-target species across various trophic levels, including algae, copepods, and mussels. Marine model organisms were selected because of the limited data available for freshwater organisms and because the marine environment serves as the ultimate receiving water body. To investigate the potential toxicity of these chemicals to human skin, in vitro toxicity tests were also conducted on human epithelial cell cultures (HaCaT cells).

## 2. Materials and Methods

### 2.1. Field Activities

#### Water Sampling

Water sampling was conducted at a wastewater collection pit located at a shelter in Mulazzo (MS), Italy, following a treatment campaign with topical ectoparasiticides. A total of 35 dogs were treated with antiparasitic products during the campaign. Specifically, 15 dogs were treated with FRONTLINE COMBO^®^ [FIP (9.8%) and (S)-methoprene (8.8%)—Boehringer Ingelheim Animal Health Italia S.p.A., Milan, Italy] at a dosage of one pipette (0.67 mL) per dog. The other 20 dogs received ADVANTIX^®^ [IMID (10%) and Permethrin (50%)–Elanco Italia S.p.A., Florence, Italy] at a dosage of one pipette (0.4 mL) per dog.

Twenty-four hours after treatment and twice daily thereafter, the dogs’ boxes were showered and cleaned to maintain the hygiene standards necessary for animal care, following the standard procedures of the shelter. The wastewater generated from the washing process was collected from the wastewater collection pit. This pit functions as a containment system, receiving water from the grates located upstream, adjacent to the dog enclosures, for further treatment. In the pit, solid materials (such as feces and fur) are separated from the liquid phase, effectively removing debris.

Sampling was performed one day before treatment (T0) and subsequently at 7, 15, 30, and 60 days post-treatment (T7–T60). At each time point, one liter of water was collected directly into sterile 1 L plastic bottles, transported to the laboratory without any pre-processing, and immediately stored at −20 °C until further analysis.

The sampling process followed the shelter’s routine wastewater collection procedures without influencing the selection or timing of treatments during the study period.

### 2.2. Laboratory Activities

#### 2.2.1. List of Reagents Used in the Laboratory Experiments

Fipronil (FIP) (batch number BCC47159), imidacloprid (IMID) (batch number BCCJ5226), fipronil (pyrazole-13C3, cyano-13C) and imidacloprid-d4, which were used as internal standards, were purchased as Supelco^®^ reagents from Merck^®^ (Milan, Italy). The reagents and materials used for in vitro tests were provided by Merck KGaA (Darmstadt, Germany) and included Dulbecco’s modified Eagle’s medium (DMEM) as the culture medium, antibiotics (penicillin and streptomycin), L-glutamine, fetal bovine serum (FBS), trypsin-EDTA solution, and phosphate-buffered saline (PBS). To assess cell viability, the MTT assay (1-(4,5-dimethylthiazol-2-yl)-3,5-diphenylformazan) was employed. For the quantification of the intracellular levels of reactive oxygen species (ROS), the fluorescent probe 2′,7′-dichlorofluorescin diacetate (DCFDA) was used. Finally, lipid peroxidation (LPO) levels were quantified using the fluorescent probe C11-BODIPY, which is specific for lipid peroxidation and was purchased from Thermo Fisher Scientific (Milan, Italy). All reagents were used following the manufacturers’ instructions to ensure the repeatability and reliability of the experiments. Sterile disposable plastic for cell culture was purchased from Sarstedt (Verona, Italy).

#### 2.2.2. Chemical Quantification of Fipronil and Imidacloprid

##### Chemical Quantification in Water Samples

Stock solutions (1 mg mL^−1^) and daily working solutions (10 and 100 ng mL^−1^) were prepared in methanol and kept at −20 °C and 4 °C, respectively.

An aliquot of 5 mL wastewater samples was added to the labeled internal standards Fipronil-(pyrazole-13C3, cyano-13C) and imidacloprid-d4 at a concentration of 5 ng mL^−1^ in the matrix. The samples were purified by using Oasis HLB SPE cartridges under vacuum. The SPE cartridges were preconditioned with 3 mL of methanol or 3 mL of Milli-Q water, and then the samples were loaded. Finally, the samples were eluted with 5 mL of methanol and collected in a 15 mL polypropylene tube. The eluate was evaporated in a rotary vacuum evaporator at 40 °C. The dried extract was reconstituted in 200 μL of methanol/water with 0.1% formic acid (60:40 *v*/*v*) and transferred to a vial.

The quantification analysis was performed by a Vanquish liquid chromatograph (Thermo Fisher Scientific, Waltham, MA, USA) coupled to an Orbitrap Exploris 120 high-resolution mass spectrometer (Thermo Fisher Scientific) equipped with a heated electrospray ionization (HESI) source. Analytes were chromatographically separated on a Raptor ARC-18 5 μm, 120 × 2.1 mm column (Restek, Bellefonte, PA, USA). The mobile phase was composed of phases A (water with 0.1% formic acid) and B (MeOH). The gradient started with 60% B, which was maintained for one minute. After six minutes, it was increased to 95% and was maintained for two minutes. After 30 s, the initial conditions were reached and maintained until the 12th minute. The flow rate was 0.3 mL min^−1^. The injection volume was 10 μL.

With respect to the detector parameters, the ion transfer tube and vaporizer temperatures were set at 330 and 280 °C, respectively, the sheath and auxiliary gas temperatures were 35 and 15 arbitrary units, and the electrospray voltages were 3.5 kV for positive mode and 3.0 kV for negative mode. The full scan acquisition was combined with a product ion scan mode for the confirmatory response, which was based on an inclusion list.

The full scan worked with a resolution of 60,000 full width at half maximum (FWHM), a scan range of 200–500 *m*/*z*, a standard gain control (AGC), an RF lens at 70%, and auto maximum injection time. The confirmatory acquisition operated at 15,000 FWHM, with a standard AGC target, auto maximum injection time, and an isolation window of 1 *m*/*z*. Fragmentation of the precursors was optimized with a two-step normalized collision energy (20 and 45 eV). The parent ion exact masses and main product ions of the compounds investigated are listed in Table S1 of the Supplementary Materials.

##### Validation of the Analytical Method

Validation was performed according to the SANTE/11312/2021 [27] Guidelines. In brief, the method was validated through the selectivity, linearity, sensitivity, recovery, precision, and evaluation of the matrix effect.

The selectivity was investigated by injecting extracted blank samples, and the absence of interferences close to the expected retention times of analytes with an S/N < 3 was verified.

The linearity was evaluated both in the solvent and via matrix-matched calibration curves, which are the last curves used to quantify real samples. It was determined by spiking both solvent and blank samples with the standard working solution in duplicate at five calibration points (0.05, 1.0, 3.0, 5.0 and 10 µg L^−1^).

The limit of quantification (LOQ) was set at the lowest level, with a recovery within the range of 70–120% and a relative standard deviation (RSD) ≤ 20%, whereas the recovery was assessed by comparing the concentrations of analytes spiked before extraction in the matrix with those spiked at the end of the extraction protocol in six replicates at the LOQ (0.05 µg L^−1^) and at 1 µg L^−1^.

The intraday repeatability was assessed through six replicates at the LOQ and at 1 µg L^−1^ and expressed as a coefficient of variation (CV%). The interday precision was evaluated in six replicates on three different working days at the same concentrations.

The matrix effect, expressed as a percentage, was calculated by comparing the peak areas of analytes spiked in blank samples after the extraction protocol to the peak areas of standards in a neat solution.

#### 2.2.3. Ecotoxicological Test Battery for Marine Species

The endpoints of the ecotoxicological assays included growth inhibition in the marine algae *Phaeodactylum tricornutum*, acute and chronic toxicity evaluations of *Acartia tonsa*, and in vitro biochemical responses of *Mytilus galloprovincialis*. These species play different ecological roles—primary producers, consumers, and filter feeders—spanning a wide range of trophic levels in aquatic ecosystems. By utilizing this combination of organisms in ecotoxicological testing, a more comprehensive understanding of the potential ecological risks associated with chemicals can be achieved, aiding in more accurate predictions of their impact on the environment and food webs. For both assays, the working concentration series was based on environmentally relevant concentrations (ERCs) of 0.03 µg L^−1^, as reported in the literature [1]. In the *M. galloprovincialis* biochemical response assay, the range of FIP and IMID concentrations was determined on the basis of the EC_x_ values derived from the previous two tests.

##### *Phaeodactylum tricornutum* Growth Inhibition

A stock solution (500 mg L^−1^) was prepared using 0.1% DMSO for FIP or ultrapure water for IMID. The working concentration series were 3.0, 0.3, 0.03, 0.003, and 0.0003 µg L^−1^ for FIP and 3000, 300, 30, 3.0, 0.3, 0.03, 0.003, and 0.0003 µg L^−1^ for IMID.

The growth inhibition of the marine algae *P. tricornutum* was evaluated following ISO 10253:2016 [28]. *P. tricornutum* Bholin (CCAP 1052/1A) was the test strain used and was purchased from CCAP (Culture Collection of Algae and Protozoa—Scottish Association for Marine Science/SAMS Research Services Ltd., Oban, UK). *P. tricornutum* algae were cultured in ASTM-enriched saltwater medium (ASTM-ESM, ASTM E1218, 2012, [29]), and working batches were prepared by inoculating 2 mL of algal culture in 20 mL of fresh ASTM medium (20 ± 2° C, under continuous illumination (6000–8000 lx)). After 72 h, the algal batches were diluted to a concentration of 106 cells mL^−1^.

For the growth inhibition bioassay, three replicates of algae were exposed to FIP and IMID concentrations for a period of 72 h at 20 ± 2 °C under continuous illumination (6000–8000 lx). The absorbance at 670 nm was measured in each well with a spectrophotometer, making use of 1 cm optic-path plastic cuvettes. The algal concentration, expressed as cells mL^−1^, was calculated from the absorbance values using the equation originally defined by Rodrigues et al. [30] and later modified by the marine research center CIBM (Livorno, Italy) group:

$$Cells\times \;{mL}^{-1}=\frac{{Abs}_{670}}{10^{-7}}$$

Potassium dichromate was used as a reference toxicant for this species, with an EC_50_ of 27.29 mg/L [95% confidence interval (CI) = 25.52 − 29.27].

##### *Acartia tonsa* Toxicity Assays

The FIP and IMID solutions were prepared using the same methodology as that of the test conducted for *P. tricornutum* at the concentrations specified (FIP: 3.0, 0.3, 0.03, 0.003, and 0.0003 µg L^−1^; IMID: 3000, 300, 30, 3.0, 0.3, 0.03, 0.003, and 0.0003 µg L^−1^). The acute and chronic toxicity of FIP and IMID were evaluated in the copepod *A. tonsa* following two different protocols:

- Acute toxicity assay: The acute toxicity of the FIP and IMID dilution series was tested in accordance with UNICHIM 2365:2012 [31]. Briefly, six replicates of 2 mL for each assessed concentration were placed in a 24-well plate. Five freshly collected fertilized *A. tonsa* eggs/nauplii were placed in each well and incubated at 20 ± 1 °C for 48 h in darkness, for a total of 30 eggs/nauplii per sample dilution. The number of immobilized individuals was counted at both 24 and 48 h. Ni^2+^ was used as the reference toxicant (EC_50_ 0.243 mg Ni^2+^ L^−1^, 95% C.I. = 0.201–0.299 mg Ni^2+^ L^−1^).
- Chronic toxicity assay: The chronic toxicity of the FIP and IMID concentration series was tested in accordance with UNICHIM 2366:2012 [32]. Briefly, three replicates of 30 mL for each assessed concentration were placed in a 50 mL glass beaker. A total of 10 freshly collected *A. tonsa* fertilized eggs/nauplii were placed in each well and incubated at 20 ± 1 °C for 7 days, with a photoperiod of 12:10 h (light:darkness). Each sample/dilution was renewed twice during the exposure (every two days). At the end of the exposure period, the number of immobilized individuals was counted. Ni^2+^ was used as a reference toxicant (EC_50_ of 0.037 mg Ni^2+^ L^−1^, 95% C.I. = 0.028–0.050 mg Ni^2+^ L^−1^).

##### In Vitro Biochemical Responses of *Mytilus galloprovincialis*

For the *M. galloprovincialis* biochemical response assay, a range of FIP and IMID concentrations was selected based on the EC_x_ values calculated from previous two tests. The selected test concentrations for both FIP and IMID were 0.1, 0.5, 1.5, 3.0, and 5.0 µg L^−1^. Adult individuals of *M. galloprovincialis* were purchased from an aquaculture farm located in the Gulf of La Spezia (NW Tyrrhenian coast, Italy) and transported to the laboratory under dry conditions. Mussels of similar size (shell lengths ranging from 60 to 70 mm) and the same sex (males) were selected.

The digestive gland and gill tissues were immediately collected separately from the mussels using sterile surgical instruments. Subsequently, subcellular S9 fractions were prepared by pooling the gills and digestive glands of 10 mussels per replicate. The excised tissues were weighed and placed into 50 mL sterile tubes. The fresh tissues were then homogenized with a phosphate buffer (50 mM potassium dihydrogen phosphate (K_2_HPO_4_) and 50 mM dipotassium phosphate (K_3_PO_4_), pH 8.00) at a ratio of 1:4 (*v*/*v*) using an Elvehjem potter. The homogenized tissues were centrifuged at 9000× *g* at 4 °C for 20 min to obtain the S9 fraction, which was then stored at −80 °C until use. S9 fractions from the gills and digestive gland were directly exposed to 0.1, 0.5, 1.5, 3.0, or 5.0 µg L^−1^ of FIP and IMID to assess the specific biochemical responses.

For each ectoparasiticide and concentration tested, six replicates, each containing 1 mg of protein, were incubated with the respective concentrations of FIP and IMID for 30 min at 25 °C. After incubation, the biochemical parameters were analyzed. Each sample was analyzed in triplicate using 96-well microplates, with a total reaction volume of 250 µL. The resulting absorbance was measured using a Synergy HT microplate reader (BioTek^®^ Inc Winooski, VT, USA), with wavelength values determined by the type of biomarker.

The total protein content was measured using the Lowry method [33]. Lipid peroxidation (LPO) was assessed by quantifying thiobarbituric acid-reactive substances (TBARS) at a wavelength of 532 nm, according to Ohkawa et al. [34]. The enzymatic activity of acetylcholinesterase (AChE) was evaluated at a wavelength of 412 nm following Ellman et al. [35]. The detailed methodology for all biomarkers can be found in Giannessi et al. [36].

#### 2.2.4. In Vitro Cell-Based Approaches

In vitro tests on cultured cells were conducted using IMID at concentrations of 62.5, 125, and 250 μg mL^−1^ and FIP at concentrations of 1.25, 2.5, and 5.0 μg mL^−1^ (with 0.1% DMSO) over a 24 h exposure period. The test solutions were prepared from a 500 mg L^−1^ stock solution in the respective medium and the potential topical effects resulting from skin exposure to both compounds were simulated.

Before each biomarker was measured, HaCaT cells [Cell Line Service (CLS, catalogue number 300493)] were cultured in a 96-well plate at a density of 1 × 10^4^ cells per well in complete medium and then incubated overnight at 37 °C and 5% CO_2_. Following incubation, the cells were exposed to various concentrations of FIP and IMID in serum-free DMEM for 24 h, with untreated cells serving as the control group (CTRL). The samples were then subjected to biomarker analyses.

##### Cell Viability Assay

At the end of the 24 h treatment, the culture medium was discarded, and 100 µL of MTT solution (0.5 mg mL^−1^ in PBS) was added to each well. The plates were subsequently incubated in the dark at 37 °C for 1 h, enabling metabolically active cells to convert MTT into formazan crystals. The formazan crystals were then dissolved via the addition of 100 μL of dimethyl sulfoxide (DMSO) to each well, with gentle shaking to ensure complete dissolution of the crystals. Optical density was then measured at a wavelength of 595 nm using a microplate reader to assess cell viability, and the resulting data are expressed as a percentage of the CTRL, as reported in the following formula:

$$Cell\;viability\left(\%\right)=100\times \frac{{OD}_{sample}}{{OD}_{control}}$$

##### Intracellular ROS Detection

Intracellular reactive oxygen species (ROS) levels were assessed via the use of the cell-permeable fluorescent probe 2,7-dichlorodihydrofluorescein diacetate (DCFDA). At the end of the 24 h treatment, a 10 μM DCFDA probe was added to each well in PBS, and the cells were incubated in the dark for 1 h at 37 °C to facilitate probe uptake and reaction with intracellular ROS. Fluorescence was subsequently measured at excitation and emission wavelengths of 485 nm and 538 nm, respectively, using a Biotek Synergy 1H plate reader (Agilent Technologies, Santa Clara, CA, USA). The ROS levels detected were normalized to cell viability, with the data expressed as a percentage with respect to the CTRL.

##### Detection of Lipid Peroxidation (LPO) Levels

The levels of lipid peroxidation (LPO) were investigated using the fluorescent probe C11-BODIPY following the manufacturer’s instructions. At the end of the 24 h treatment, a 1 μM C11-BODIPY solution in PBS was added to each well, and the cells were incubated in the dark for one hour at 37 °C. Fluorescence was subsequently measured using a Biotek Synergy 1H plate reader (Agilent Technologies, Santa Clara, CA, USA). The excitation and emission wavelengths used were 488 nm and 510 nm, respectively. The detected LPO levels were normalized to cell viability, and the untreated sample was used as a control. The data obtained are expressed as percentages with respect to the CTRL.

### 2.3. Statistical Analyses

The assumptions of normality and homogeneity of variance were tested via the Shapiro-Wilk test and Bartlett test, respectively. The results indicated that the data did not follow a normal distribution.

Statistical analyses of *Phaeodactylum tricornutum* growth inhibition and *Acartia tonsa* toxicity results were performed using the freeware tool MOSAIC (https://mosaic.univ-lyon1.fr/, accessed on 24 April 2025), developed by Université Claude Bernard Lyon 1 for ecotoxicological modeling.

EC_10/20/50_ values were calculated using the MOSAICbioacc module by fitting a dose-response model (3-parameter log-logistic model) through Bayesian inference. The model provided EC_10/20/50_ estimates along with 95% confidence intervals, and model fit was assessed with integrated diagnostic tools.

The effects of different concentrations of each ectoparasiticide were evaluated via one-way ANOVA, followed by Tukey’s post hoc test (*p* ≤ 0.05) for multiple comparisons. Graphs and statistical analyses were generated using GraphPad Prism 9.5.1^®^ (www.graphpad.com, accessed on 20 April 2025).

To compare the effects of FIP and IMID at each concentration on the biomarkers of interest in each *M. galloprovincialis* tissue (LPO levels and AChE activity in the gills and digestive glands), two-way ANOVA was applied, followed by Tukey’s post hoc test (*p* ≤ 0.05) for multiple comparisons. Graphs and statistical analyses were generated using GraphPad Prism 9.5.1^®^ (www.graphpad.com, accessed on 20 April 2025).

For HaCaT cultured cells, the effects of different concentrations of each ectoparasiticide on various biomarkers (MTT, ROS production, and LPO levels) were evaluated via one-way ANOVA, followed by Tukey’s post hoc test (*p* ≤ 0.05) for multiple comparisons. Graphs and statistical analyses were generated using GraphPad Prism 9.5.1^®^ (www.graphpad.com, accessed on 20 April 2025).

## 3. Results

### 3.1. Fipronil and Imidacloprid Concentrations in Wastewater

**Figure 1:**
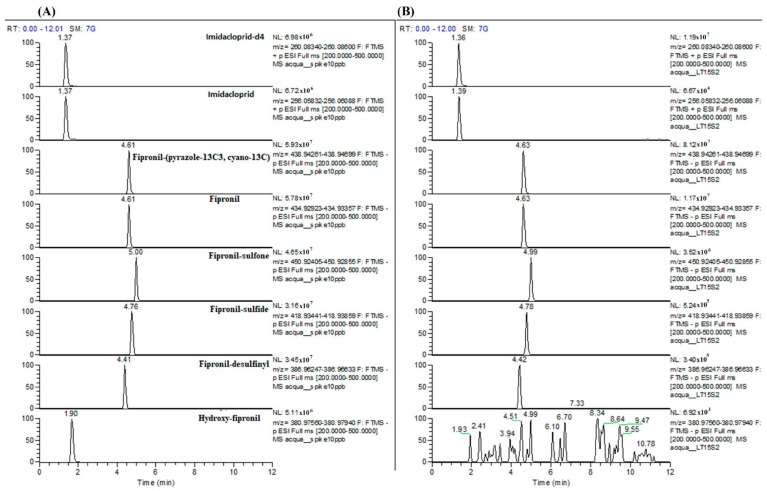
Extracted full scan chromatograms of the internal standards and all analytes at 10 µg L^−1^ in matrix (**A**) in comparison with a positive sample (**B**).

The concentrations of IMID, FIP, and FIP-sulfone in the wastewater samples remained stable for at least 60 days after the animals were treated, with average values of 0.18, 0.50 and 0.20 µg L^−1^, respectively (Table 1). Other metabolites investigated, such as FIP-sulfide, FIP-desulfinil, and hydroxy-FIP, were found at concentrations < 0.07 µg L^−1^ or below the LOQ. In Figure 1, the extracted full scan chromatograms of the internal standards and all analytes at 10 µg L^−1^ in matrix (A) in comparison with a positive sample (B) are reported as an example.

### 3.2. Validation of the Analytical Method for Fipronil and Imidacloprid

The analytical method was validated in terms of selectivity, linearity, sensitivity, recovery, precision, and evaluation of the matrix effect. The validation data are reported in Table 2.

### 3.3. Ecotoxicological Test Battery Responses

#### 3.3.1. *Phaeodactylum tricornutum* Growth Inhibition

No significant effects were detected in the growth inhibition assay for *P. tricornutum* at any of the tested FIP or IMID concentrations (Tables S1 and S2).

#### 3.3.2. *Acartia tonsa* Toxicity Assays

No significant effects were detected in the *A. tonsa* acute toxicity assay compared with the control at both 24 and 48 h of FIP and IMID exposure (Table S3).

In contrast, a significant reduction in naupliar mobility (*p* < 0.001) was observed for both FIP and IMID after 7 days of exposure at concentrations of 0.003, 0.03, 0.3, and 3.0 µg L^−1^. This led to the calculation of EC_10_ and EC_20_ values of 1.7 (0.06–6.59) and 2.8 (0.436–8.51) µg L^−1^, respectively, for FIP and EC_10_, EC_20_ and EC_50_ values of 2.59 (0.80–6.33), 7.60 (3.12–15.8) and 47.50 (25.30–90.10) µg L^−1^, respectively, for IMID (Table 3).

#### 3.3.3. Biochemical Markers of *Mytilus galloprovincialis*

**Figure 2:**
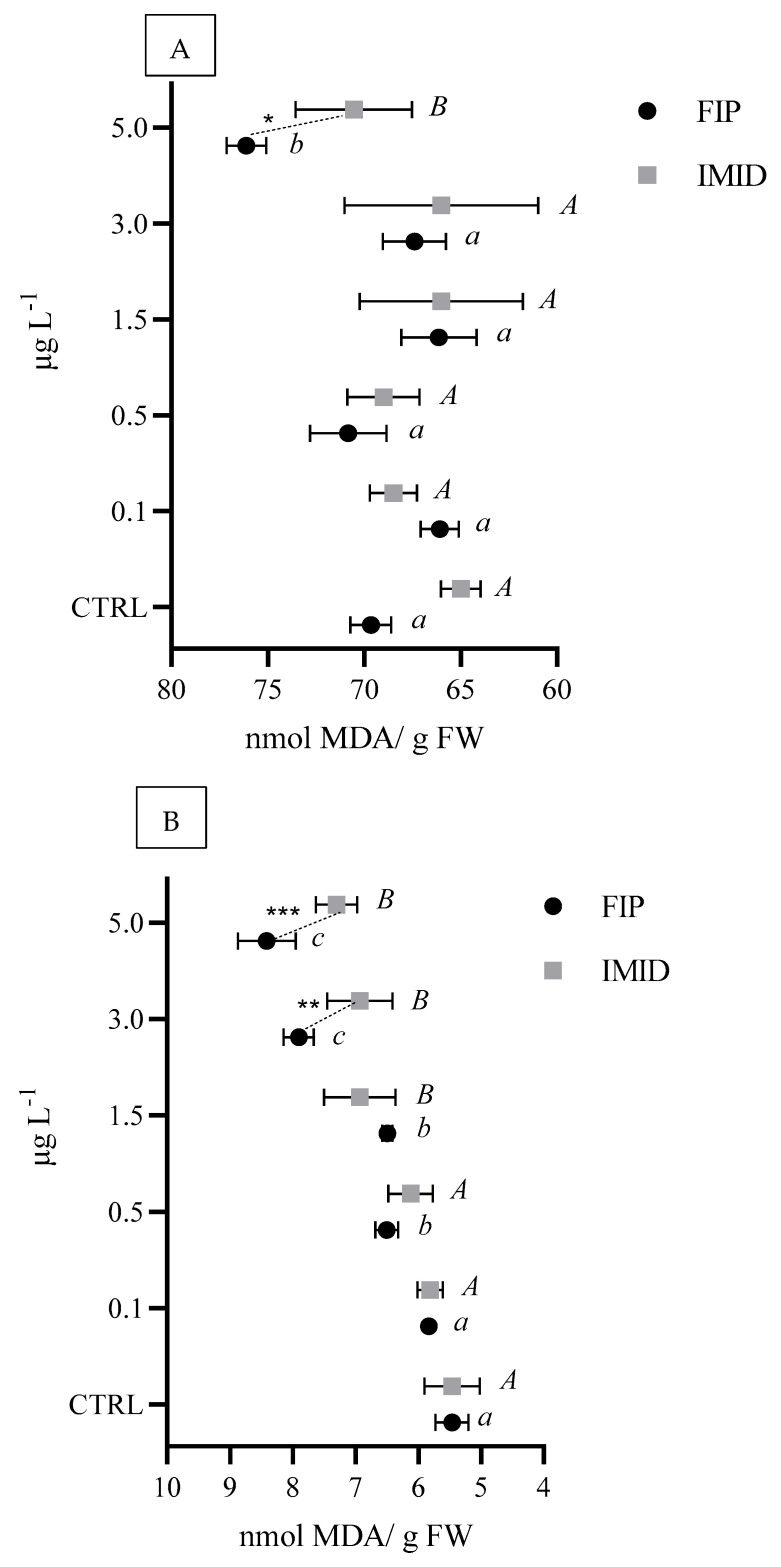
Lipid peroxidation levels (LPO–nmol MDA/g fresh weight (FW)) in *M. galloprovincialis* digestive glands (**A**) and gills (**B**) exposed to a range of 0.1–5.0 µg L^−1^ FIP or IMID. Different letters represent statistical differences among concentrations of each ectoparasiticide (*A* and *B* = *p* < 0.05; *a*, *b* and *c* = *p* < 0.01), while asterisks indicate significant differences between ectoparasiticides at each tested concentration (* *p* < 0.05; ** *p* < 0.01; *** *p* < 0.001).

A significant increase in LPO levels was observed in the digestive gland after exposure to 5 µg L^−1^ of both FIP and IMID, with significantly higher LPO levels with FIP than with IMID exposure (Figure 2A). The increase in LPO was more pronounced in the gills than in the digestive glands, with a significant increase in the levels starting at 0.5 µg L^−1^ for both FIP and IMID. Compared with IMID, FIP caused significantly greater damage at both 3.0 µg L^−1^ and 5.0 µg L^−1^ (Figure 2B).

**Figure 3:**
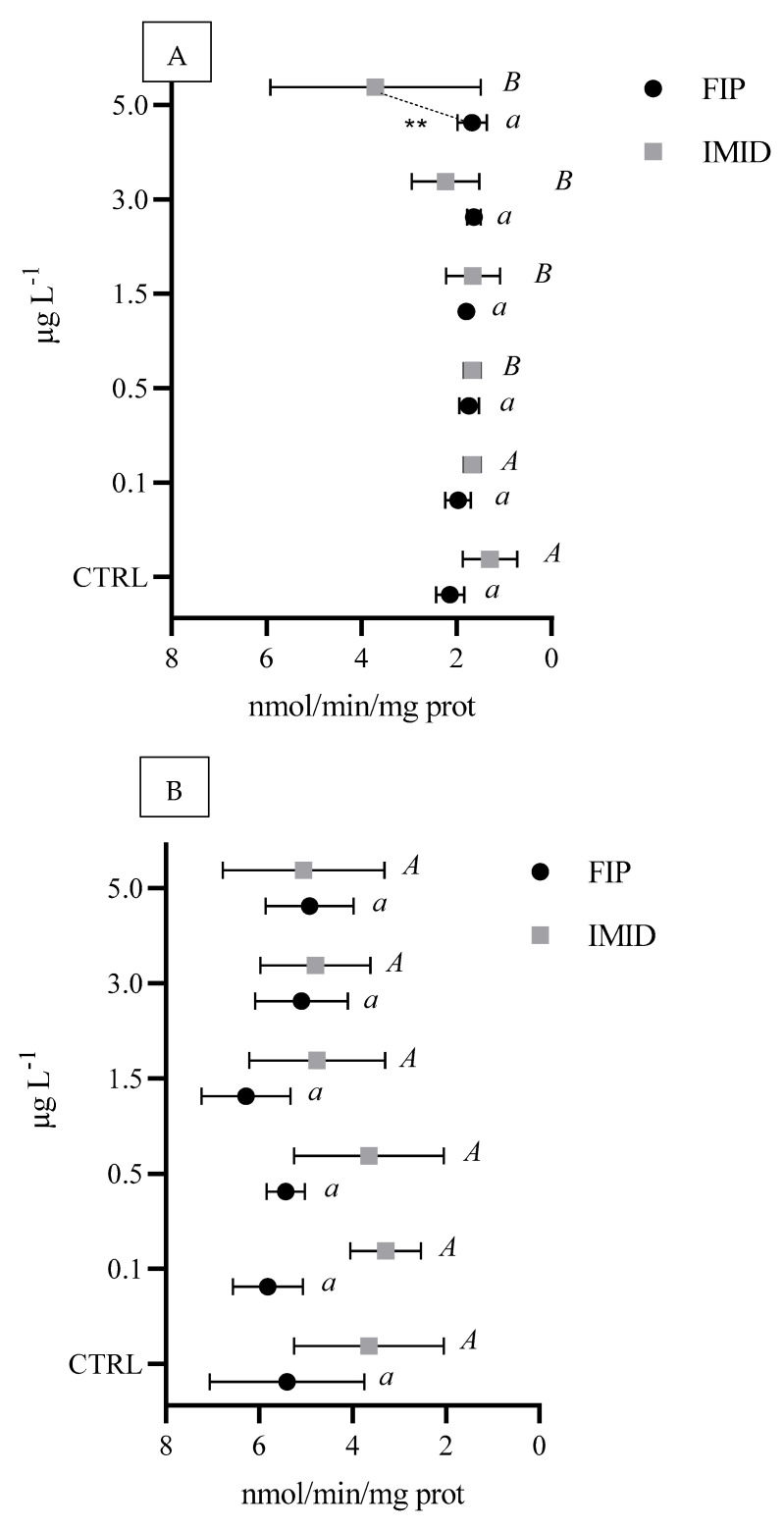
Enzymatic activity of acetylcholinesterase (nmol/min/mg of protein) in *M. galloprovincialis* digestive glands (**A**) and gills (**B**) exposed to a range of 0.1–5.0 µg L^−1^ FIP or IMID. Different letters represent statistical differences among concentrations of each ectoparasiticide (*A* and *B* = *p* < 0.05; *a*, = *p* < 0.01) Asterisks indicate significant differences between ectoparasiticides at each tested concentration (** *p* < 0.01).

A significant increase in AChE activity (*p* < 0.05) was observed only in the digestive gland exposed to IMID in the range of 0.5–5.0 µg L^−1^ (Figure 3A). No alterations in AChE activity were detected in the gills exposed to both ectoparasiticides (Figure 3B).

### 3.4. In Vitro Cell-Based Responses

**Figure 4:**
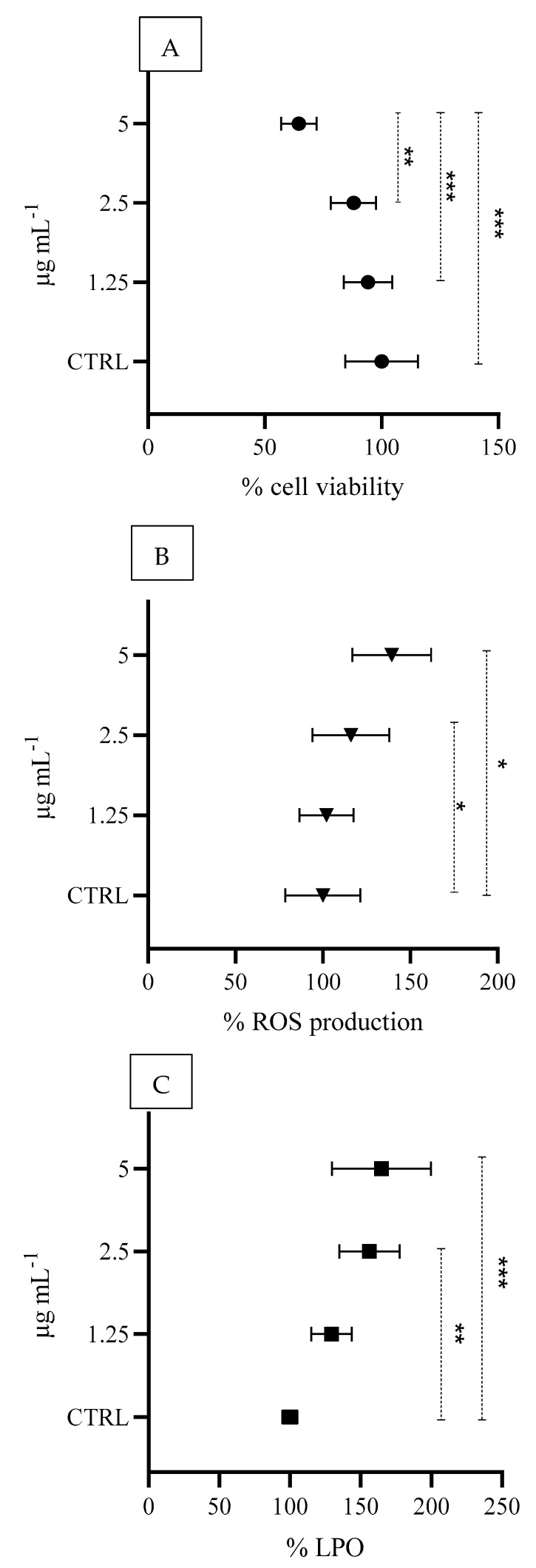
Percentage of cell viability (MTT assay) (**A**), percentage of reactive oxygen species production (**B**), and percentage of lipid peroxidation (LPO), and (**C**) HaCaT cultured cells exposed to FIP. Asterisks indicate significant differences among concentrations for each ectoparasiticide tested (* *p* < 0.05; ** *p* < 0.01; *** *p* < 0.001).

A significant concentration-dependent reduction in cell viability (% MTT) of 94.2%, 88.0% and 64.6% was observed at FIP concentrations of 5.0, 2.5 and 1.25 μg mL^−1^, respectively (Figure 4A). Compared with those in the control, significant increases in ROS production and LPO levels were detected at FIP concentrations of 2.5 and 5 μg mL^−1^ (Figure 4B,C).

**Figure 5:**
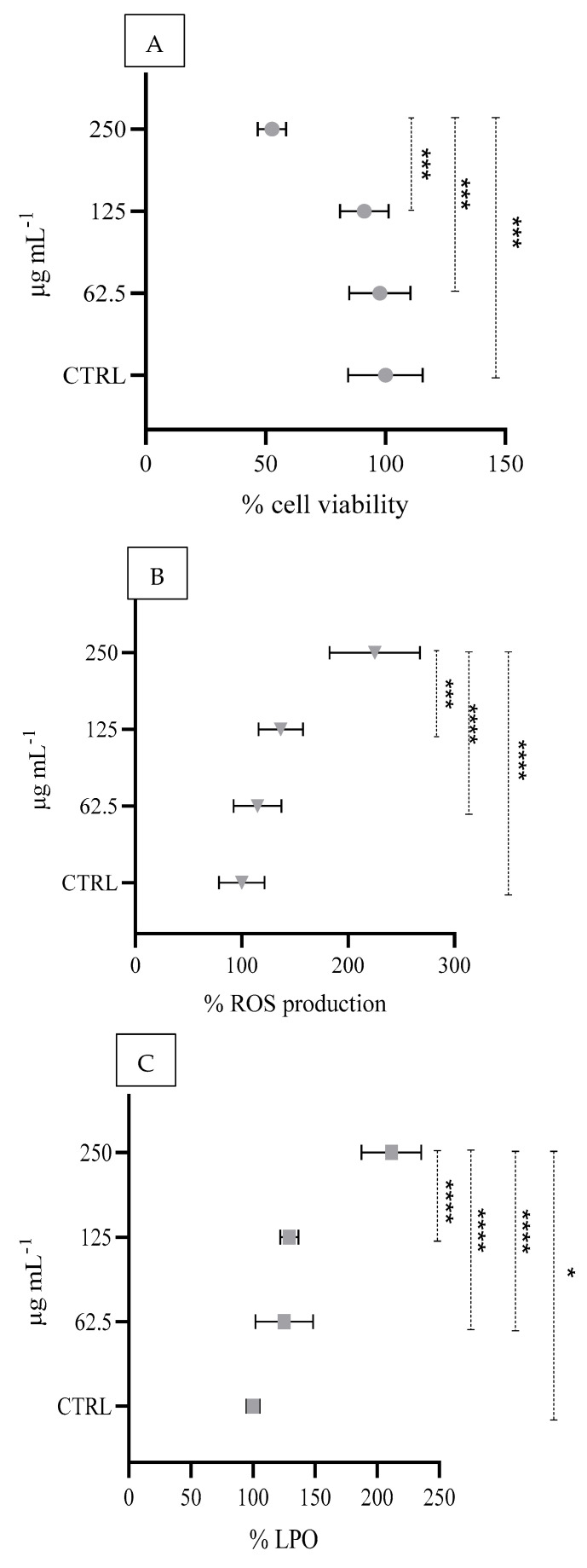
Percentage of cell viability (MTT assay) (**A**), percentage of reactive oxygen species production (**B**), and percentage of lipid peroxidation (LPO), and (**C**) HaCaT cultured cells exposed to IMID. Asterisks indicate significant differences among concentrations for each ectoparasiticide tested (* *p* < 0.05; *** *p* < 0.001; **** *p* < 0.0001).

A significant reduction in cell viability of 97.7, 91.2 and 52.6% was observed at 250, 125 and 62.5 μg mL^−1^ concentrations of IMID, respectively (Figure 5A), along with an increase in ROS production (Figure 5B) and LPO levels (Figure 5C) compared with those of the control.

Figure S1 presents cell morphology images of the control alongside those exposed to the highest concentrations of IMID and FIP.

## 4. Discussion

### 4.1. Fipronil and Imidacloprid Concentrations in Wastewater

Although the water quantification in our study was limited to the wastewater outflow area of the shelter, where wastewater is specifically generated by routine pet and box care activities, our results provide a realistic scenario for assessing how these chemicals enter the environment through everyday pet care practices. The results can also be used for monitoring the stability of compound concentrations over time.

Compared with the literature, our data highlight that the concentrations of IMID, FIP and FIP metabolites were significantly higher—between 10 and 100 times greater—than those reported in European studies of freshwater surface waters. The average IMID concentrations in freshwater, reported by the ECHA, range from 0.01 to 0.03 µg L^−1^, which are notably lower than the concentrations observed in our study (0.18 µg L^−1^).

In contrast to freshwater environments, data on IMID concentrations in marine waters are less prevalent. However, Liu et al. [37] reported that IMID concentrations in marine waters ranged from undetectable to 0.636 µg L^−1^, with a median concentration of 0.00414 µg L^−1^.

Both FIP and IMID remained stable in water for at least 60 days, confirming their persistence in aqueous environments, as shown by Perkins et al. [16,17]. Data reported by the EMA [1] indicate that IMID, FIP and their metabolite concentrations typically remain below risk thresholds in environmental settings. However, the concentrations found in our study—FIP at 0.50 µg L^−1^ and FIP-sulfone at 0.20 µg L^−1^—were significantly higher than those typically reported in the EMA’s environmental data.

### 4.2. Ecotoxicological Assessment

In the present study, the endpoints for the marine ecotoxicological assays included growth inhibition of the marine algae *P. tricornutum*, acute and chronic toxicity assessments of the marine copepod *A. tonsa*, and in vitro biochemical responses of the marine mussel *M. galloprovincialis*. These species represent distinct ecological functions—primary producers, consumers, and filter feeders—covering a broad range of trophic levels in marine ecosystems. The use of this combination of organisms in ecotoxicological testing provides a more comprehensive understanding of the potential ecological risks posed by chemicals, facilitating better predictions of their impact on the environment and food webs.

Our decision to use non-target marine species as model organisms was a result of the scarcity of available data in the literature for marine environments in contrast to the more extensive studies conducted on freshwater species. Additionally, the marine environment represents the ultimate receiving body for many pollutants, making it crucial to assess the potential ecological risks in these ecosystems.

From an ecotoxicological perspective, FIP concentrations in European freshwaters are generally lower than 0.05 µg L^−1^, whereas ecotoxicological effects have been observed at 0.50 µg L^−1^ [38]. In our study, toxicological assessments using *M. galloprovincialis* demonstrated that FIP and IMID, at concentrations of 0.50 and 1.50 µg L^−1^, respectively, increased the levels of LPO in both the gills and digestive glands. Compared with the literature, the only study available on mussels reported that sublethal concentrations of FIP induced oxidative stress and histopathological damage in *Unio delicatus* [39]. In another model species, the fish *Cyprinus carpio*, FIP also led to increased LPO levels of 0.65 mg L^−1^ over exposure periods of 7, 30, and 90 days [40].

Similarly, IMID has been shown to increase LPO levels in the brain tissue of the neotropical fish *Prochilodus lineatus* [41] and in the gills of the freshwater mussel *Unio mancus* [42]. In our study, IMID increased ACh enzyme activity in the mussel digestive gland.

Recent studies have emphasized the growing concern regarding neonicotinoid contamination in aquatic environments and its potential effects on nontarget organisms, such as bivalves. Pagano et al. [43] reviewed the impact of neonicotinoids, including IMID, on aquatic invertebrates, with a specific focus on *M. galloprovincialis*. Pagano’s review highlights various biochemical effects of neonicotinoids, including alterations in enzyme activity and potential impacts on mussel health due to exposure to environmentally relevant concentrations. Similarly, Kuchovská et al. [44] examined the effects of IMID and other substances on the Pacific oyster (*Magallana gigas*), revealing how neonicotinoids influence bivalves and how their gene expression is associated with toxicity. Together, these findings, along with our findings, confirm the significant impact of FIP and IMID on non-target aquatic organisms at different trophic levels and highlight the need for further investigation into their ecological effects.

EC_x_ and LC_x_ values are essential for evaluating the toxicity of chemicals in aquatic ecosystems and organisms. In the literature, the LC_50_ values for FIP range from 2.18 to 19.12 μg L^−1^ when different model species are used [45,46]. Our findings are consistent with these values, with EC_10_ and EC_20_ values of 1.7 µg L^−1^ and 2.8 µg L^−1^, respectively, for *A. tonsa* exposed to FIP. When comparing our marine model organism to other freshwater crustacean species, *A. tonsa* displayed greater sensitivity to FIP than *Ceriodaphnia dubia* [47,48], *Daphnia magna* [49,50], and *Simocephalus elizabethae* [51], exhibiting LC_50_ values ranging from 0.98 to 19.12 μg L^−1^. These findings suggest that marine crustaceans may be more vulnerable to certain contaminants, such as FIP, potentially due to differences in environmental stressors, osmoregulatory processes, or adaptive mechanisms between marine and freshwater species.

Most studies have focused only on freshwater settings, creating a substantial gap in knowledge about the effects of FIP in saline or brackish environments. This lack of data is particularly relevant to our findings, as toxicity mechanisms and bioavailability may differ in marine environments, highlighting the need for further research to clarify FIP’s ecological impacts across various aquatic ecosystems.

Similarly, in invertebrates, IMID causes significant sublethal effects [52,53,54]. Our study revealed EC_10_ and EC_20_ values of 2.59 µg L^−1^ and 7.6 µg L^−1^, respectively, for *A. tonsa* following chronic exposure, which are consistent with the values reported in the literature. In a similar study, Picone et al. [55], for example, reported an EC_50_ of 8.84 μg L^−1^ for the same species. Together with the literature, our findings demonstrate that the calculated ECx values exceed the European Commission’s proposed standards for long-term exposure (8.3 ng L^−1^) [56]. These results suggest that current regulations may have underestimated the potential long-term effects of IMID on marine invertebrates.

### 4.3. In Vitro Cell-Based Toxicity

HaCaT cells, a human keratinocyte cell line derived from normal skin, are well suited for testing antiparasitic toxicity due to their similarity to native keratinocytes given that they preserve essential features such as proliferation and differentiation. These cells demonstrate functional competence in critical cellular processes, including cytotoxicity and inflammation, making them valuable for comprehensive drug evaluations. In our study, the potential cytotoxicity of FIP and IMID in HaCaT human keratinocytes was investigated simulating acute exposure. The analysis of cellular responses to FIP and IMID revealed that both chemicals triggered oxidative stress mechanisms, as evidenced by high levels of ROS and LPO, which ultimately led to reduced cell viability in exposed skin cells.

Regarding IMID exposure, our concentration range (62.5, 125 and 250 μg mL^−1^) was based on cytotoxicity data reported in the literature using the same cell line. For example, Leri et al. [57] and Silva et al. [58] reported oxidative stress responses in HaCaT cells exposed to IMID at concentrations of 200 μg mL^−1^. IMID-induced cytotoxicity has also been reported in other cell lines, including Caco-2, HepG2, A431, SK-MEL-5, and RAW 264.7, with IC_50_ values exceeding 200 μg mL^−1^, further supporting its potential cytotoxic effects at the cellular level.

Similarly, we selected FIP concentrations based on direct evidence indicating that FIP exposure can cause skin irritation in humans [59]. Although no studies have specifically reported oxidative stress responses in keratinocyte cultures, exposure to 40 μg mL^−1^ FIP in SH-SY5Y human neuronal cells induced cytotoxic effects. Furthermore, a concentration range of 20–80 μg mL^−1^ resulted in growth inhibition in human epithelial Caco-2 cells and reduced MTT activity [60]. Notably, the concentrations tested in the present study (0.25, 2.5, and 5.0 μg mL^−1^) were up to two times lower than those reported in previous studies that induced cytotoxicity in various cell models. These findings suggest that even at lower concentrations, FIP may exert toxic effects, potentially through oxidative stress mechanisms, thus reinforcing the need for further investigation into its impact on human skin cells. Overall, assessing their impact on keratinocyte function and skin barrier integrity could offer valuable insights into their potential dermatotoxicity. This knowledge would support the implementation of stricter preventive measures and strengthen the importance of adhering to usage guidelines to minimize health risks for humans.

## 5. Conclusions

Our analysis of wastewater samples from the Italian shelter provided valuable insights into the concentrations of IMID, FIP, and their metabolites in untreated water, establishing a baseline for effluent flowing directly from the facility. Our results indicated that these compounds remained stable for up to 60 days post-treatment, suggesting significant environmental persistence. Ecotoxicological tests on marine organisms revealed toxic effects of both FIP and IMID in copepods and bivalves, with EC_10_ and EC_20_ levels that could harm aquatic life, especially with prolonged exposure. Biochemical damage in mussel gills was observed at FIP concentrations as low as 0.5 µg L^−1^ and IMID at 1.5 µg L^−1^, emphasizing the vulnerability of marine species to these compounds. Furthermore, mussels emerged as a valuable model species for studying the impact of these contaminants, contributing crucial new insights to the literature.

However, no significant effects were observed in the growth inhibition assay for *P. tricornutum* at any of the concentrations tested for FIP or IMID. Previous studies have suggested that algae may possess inherent resistance or differences in metabolic pathways that reduce their sensitivity to these compounds [61,62].

Our findings are consistent with those outlined in EMA documents, underscoring the urgent need for effective monitoring and preventive strategies. This is especially important considering the growing population of companion animals and evolving management practices, which contribute to the increasing environmental burden through the widespread use and potential accumulation of ectoparasiticides in ecosystems.

Strengthening regulatory frameworks and promoting sustainable alternatives could mitigate the risks associated with the environmental persistence and toxicity of these substances. The in vitro cytotoxicity results for FIP and IMID further emphasize the need for strict adherence to usage instructions to minimize health risks to humans. Overall, these results highlight the interconnected impacts of ectoparasiticide treatments on environmental, animal, and human health, supporting the need for a One Health approach to assess, regulate, and mitigate the risks associated with their use.

## Figures and Tables

**Table 1:** Concentrations of imidacloprid (IMID), fipronil (FIP) and their metabolites (mean ± sd) in wastewater samples.

	µg L^−1^					
Sample	IMID	FIP	FIP-Sulfone	FIP-Sulfide	FIP-Desulfinil	Hydroxy-FIP
T0	n.d.	<LOQ	0.05	n.d.	n.d.	n.d.
T7	0.36	0.42	0.10	<LOQ	<LOQ	n.d.
T15	0.06	1.36	0.40	0.07	0.05	n.d.
T30	0.08	0.16	0.31	<LOQ	0.06	n.d.
T60	0.20	0.05	0.13	<LOQ	<LOQ	n.d.
mean	0.18	0.50	0.20	-	0.06	-
sd	0.12	0.52	0.13	-	0.01	-

n.d.: Not detected; LOQ: 0.05 µg L^−1^; LOD: 0.01 µg L^−1^.

**Table 2:** Validation results for the LC-HRMS analytical protocol.

Analytes	Validation Levels (µg L^−1^)	Recovery * %	CV * Intraday %	CV * Interday %	Linearity R^2^	Matrix Effect %
IMID	LOQ 0.05 1.0	99 100	5 4	7 6	0.9997	98
FIP	LOQ 0.05 1.0	99 100	5 3	7 5	0.9994	98
FIP-sulfide	LOQ 0.05 1.0	98 101	5 3	7 6	0.9991	97
FIP-sulfone	LOQ 0.05 1.0	97 99	5 3	8 6	0.9993	97
FIP-desulfinyl	LOQ 0.05 1.0	97 100	6 4	9 7	0.9995	96
Hydroxy-FIP	LOQ 0.05 1.0	96 98	9 6	11 8	0.9990	94

* Both recovery and precision were evaluated at LOQ and 1 µg L^−1^ in six replicates.

**Table 3:** ECx values (95% confidence intervals) of *A. tonsa* nauplii mobility after 7 days of FIP and IMID exposure.

ECx	µg L^−1^ (95% C.I.)	
	FIP	IMID
EC_10_	1.70 (0.06–6.59)	2.59 (0.80–6.33)
EC_20_	2.80 (0.436–8.51)	7.60 (3.12–15.8)
EC_50_	-	47.50 (25.30–90.10)

## Data Availability

The original contributions presented in this study are included in this article. Further inquiries can be directed to the corresponding author.

## Supplementary Materials

The following supporting information can be downloaded at: https://www.mdpi.com/article/10.3390/ani15111533/s1, Table S1: Algal concentration ± standard deviation; mean growth rate (µ) and percentage of growth rate inhibition with respect to control (I%) of *P. tricornutum* after 72 ± 2 h of fipronil exposure; Table S2: Algal concentration ± standard deviation; mean growth rate (µ) and percentage of growth rate inhibition with respect to control (I%) of *P. tricornutum* after 72 ± 2 h of imidacloprid exposure; Table S3: Percentage of immobilization (and relative standard deviation) of *A. tonsa* nauplii after 24 and 48 h of exposure to fipronil and imidacloprid. Figure S1: Phase-contrast images of HaCaT cultured cell morphology: (A) control (90% confluence); (B) exposed to 5 µg mL^−1^ FIP; (C) exposed to 250 µg mL^−1^ IMID, captured using a Leica Microsystems Model (Mateo TL, Wetzlar, Germany) microscope with a 4X objective.
