# Supplementary material for: Environmental Risks and Toxicity of Fipronil and Imidacloprid Used in Pets Ectoparasiticides
Source: Animals (Basel). 2025 May 23;15(11):1533. doi: 10.3390/ani15111533 (PMC12153832; doi:10.3390/ani15111533)
Supplement: Supplementary file 1 [file animals-15-01533-s001.zip › animals-3594553-supplementary.pdf]

Supplementary Materials

Table S1: Algal concentration ± standard deviation; mean growth rate (μ) and percentage of growth rate inhibition with respect to control (I%) of *P. tricornutum* after 72 ± 2 hours of fipronil exposure.

$\mu g\ L^{-1}$	Cell mL^{-1} ± S.D.			μ	I%
0 (CTRL)	603333	±	46332	1.37	
3.0	570000	±	26458	1.35	1.39
0.3	553333	±	11547	1.34	2.11
0.03	563333	±	15275	1.34	1.67
0.003	583333	±	15275	1.36	0.82
0.0003	573333	±	15275	1.35	1.24

Table S2: Algal concentration ± standard deviation; mean growth rate (μ) and percentage of growth rate inhibition with respect to control (I%) of *P. tricornutum* after 72 ± 2 hours of imidacloprid exposure.

$\mu g\ L^{-1}$	Cell mL^{-1} ± S.D.			μ	I%
0 (CTRL)	603333	±	46332	1.37	
3000	380000	±	10000	1.21	6.29
300	433333	±	15275	1.26	2.90
30	446667	±	5774	1.27	2.12
3	436667	±	5774	1.26	2.70
0.3	446667	±	15275	1.27	2.12
0.03	466667	±	15275	1.28	0.99
0.003	453333	±	20817	1.27	1.74
0.0003	480000	±	17321	1.29	0.27

Table S3: Percentage of immobilization (and relative standard deviation) of *A. tonsa* nauplii after 24 and 48 h of exposure to fipronil and imidacloprid.

$\mu g\ L^{-1}$	Immobilization (%) ± S.D. 24h		Immobilization (%) ± S.D. 48h	
	fipronil	imidacloprid	fipronil	imidacloprid
0 (CTRL)	3.33 ± 0.41	3.33 ± 0.41	20.00± 0.00	20.00± 0.00
3000	-	6.62 ± 0.50	-	26.67± 0.53
300	-	6.62 ± 0.50	-	26.67± 0.53
30	-	3.33 ± 0.41	-	20.67± 0.54
3	3.33 ± 0.41	3.33 ± 0.41	20.00± 0.89	20.00± 0.00
0.3	0.00 ± 0.00	3.32 ± 0.00	26.67± 0.00	16.67± 0.75
0.03	6.67 ± 0.52	3.33 ± 0.41	23.33± 0.75	3.33± 0.75
0.003	6.67 ± 0.52	3.33± 0.41	16.67± 0.75	3.33± 0.75
0.0003	3.33 ± 0.41	3.33 ± 0.41	3.33± 1.83	3.33± 0.75

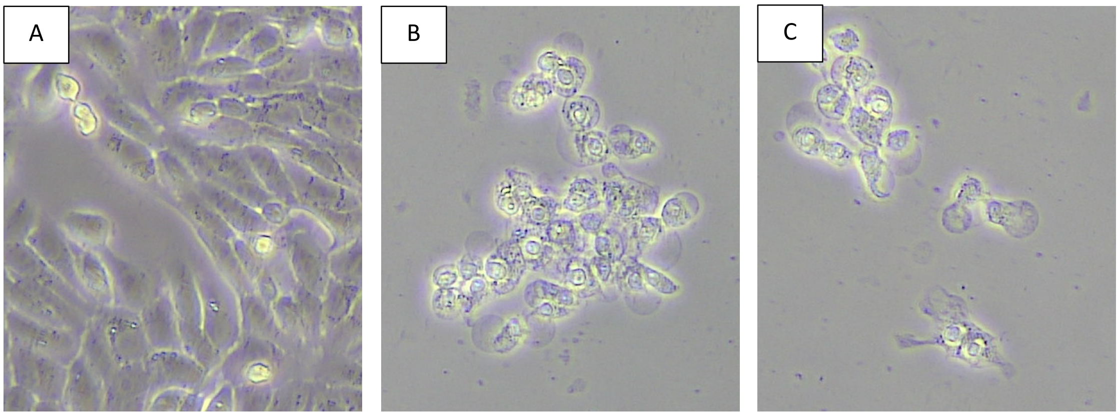

Figure S1: Phase-contrast images of HaCaT cultured cell morphology: A) control (90% confluence); B) exposed to 5 µg mL^{-1} FIP; C) exposed to 250 µg mL^{-1} IMID, captured using a Leica Microsystems Model (Mateo TL) microscope with a 4X objective.
